# Iodine a Preventive of Mammary Abscess

**Published:** 1853-12

**Authors:** H. C. Stewart

**Affiliations:** Salisbury, Pa.


### *Iodine a preventive of Mammary Abscess.*

BY H. C. STEWART, M. D., SALISBURY, PA.

This communication, I presume, will fall under the notice of no physician unacquainted with what is usually termed mammary abscess, both as regards the condition of the breast and the best known means of obviating that distressing condition to which the "lying in woman" is so often subjected.

Perhaps no organ of the body is capable of producing a greater amount of suffering to the patient, and vexation to the physician, than the female breast. Situated upon a prominent part of the body—delicately constructed—influencing and being influenced at times by other organs, it is adapted to the performance of an important function, the disturbance of which must necessarily produce a disagreeable and dangerous result, often requiring the best efforts of the physician to counteract.

Seeing, then, that these are so, we have been lead to inquire, Is there no remedy? or must our patients, after having undergone the agony of parturition, still suffer on simply because their breasts have not been properly and sufficiently relieved of milk as fast as secreted? If mammary abscess cannot be prevented, it is not because *remedies* have not been proposed for it; for amongst all the "ills that flesh is heir to," there is none, perhaps, for which such a multitudinous variety of *cures* has been tried. This is probably the best evidence of the difficulty of preventing such an occurrence.

The first indication that suggests itself to the mind of the physician, is to remove the tension by withdrawing the milk. But this cannot always be done; for in how many cases do we find a complete obstruction of the ducts; others, again, where there are no nipples, consequently no outlet for the milk. Have we no remdy here, or must we let the gland inflame and then bleed and apply leeches and "poultices to favor suppuration." and when the abcess forms, open it with a lancet, and run the risk of forming a milk fistula, then apply adhesive strips, and if all this fail—*let it alone?*

In the early part of my practice I was called to attend a lady, the mother of five children, none of whom she had ever suckled, owing to an inversion of the nipples, and consequent obstruction of the ducts. So thorough was this obstruction, that the best efforts of the physicians, on former occasions, had totally failed to relieve the breasts of a particle of milk; consequently the woman had suffered on every occasion from mammary abscess.

In giving me a history of the treatment at different times, she said at one time she came near losing both breasts; when the physician, (dead at the time of this conversation,) as a last remedy, applied something, which, from the description given me, I believe to be iodine. Knowing the efficacy of that in all glandular affections, I resolved to try it as soon as the breasts showed any signs of inflammation. On the third day, finding them large, heavy and intensely painful, I

made an application to the breasts of iodine ointment spread upon linen, which gave almost immediate relief. After a few applications I found the breasts "perfectly flaccid, completely cool, and admitting of the freest palpation and handling, without the woman making any complaint." From the favorable result in the above case, I was induced to try it in two similar cases, with the same success, and, so far as I know, it never failed in the hands of any of my medical friends to whom I have recommended it, and not a few there are who can bear testimony to its virtues.

With these few suggestions, I respectfully submit it to the profession, hoping that it may not disappoint their expectations.—*Medical Examiner.*

